# Evaluating sustainability in the Childhood Obesity Research Demonstration project: the model and process

**DOI:** 10.1186/s13690-020-0397-2

**Published:** 2020-02-13

**Authors:** Rebecca E. Lee, Dennis Kao, Nathan H. Parker, Allen M. Hallett, Camila Y. Kochi, Maria J. Modelska, Hanadi S. Rifai, Daniel P. O’Connor

**Affiliations:** 1 Center for Health Promotion and Disease Prevention, Edson College of Nursing and Health Innovation, Arizona State University, 550 N. 3rd St, Phoenix, AZ 85004 USA; 2 School of Social Work, Carleton University, 509 Dunton Tower, 1125 Colonel By Drive, Ottawa, ON K1S 5B6 Canada; 3 Department of Behavioral Science, University of Texas MD Anderson Cancer Center, Unit 1330, CPB3.3278, PO Box 301439, Houston, TX 77230 USA; 4 Department of Epidemiology, Human Genetics and Environmental Sciences, The University of Texas Health Science Center at Houston (UTHealth) School of Public Health in Austin, 1616 Guadalupe St, Ste 6.300, Austin, TX 78701 USA; 5 Department of Pharmacological & Pharmaceutical Sciences, University of Houston, 4849 Calhoun Rd. #5007A, Houston, TX 77204 USA; 6 Department of Civil and Environmental Engineering, Cullen College of Engineering, University of Houston, 4726 Calhoun, N107 Engineering Bldg 1, Houston, TX 77204 USA; 7 Department of Health and Human Performance, HEALTH Research Institute, University of Houston, 3875 Holman St. Rm. 104 Garrison, Houston, TX 77204 USA

**Keywords:** Community, Organization, Children, Intervention study, Physical activity, Diet

## Abstract

**Background:**

In the context of health-related interventions, sustainability is the capacity to maintain the changes resulting from the intervention. These can be improved policies, practices or trends intended to improve population health. The Childhood Obesity Research Demonstration (CORD) project was a multi-site, multi-intervention collaboration testing the Obesity Chronic Care Model with interventions for childhood obesity prevention and management. We present the model, definitions and methodology used for the cross-site sustainability evaluation of CORD.

**Methods:**

We applied the Ecologic Model of Obesity to childhood obesity interventions to operationalize four sustainability constructs: replicability, continuation of benefits, institutionalization, and community capacity. We used a triangulation approach and employed mixed methods to assess sustainability constructs at each level of the Ecologic Model of Obesity: Micro, Meso, Exo and Macro. We constructed checklists to count and code intervention activities, use of evidence-based practices among providers, and environmental factors and policies hypothesized to influence intervention sustainability. We developed in-depth interviews for principal investigators and project leads. We applied the Wilder Collaboration Factors Inventory with key stakeholders.

**Results:**

Lessons learned suggested that sustainability constructs should be clearly identified and operationalized a priori. Constructs must be flexible to account for differences between intervention plans and implementation to obtain robust and informative data.

**Conclusion:**

Strong links are needed among researchers, program implementers and communities to accomplish consistent, robust and valuable data collection efforts to assure sustainable and healthy communities.

## Background

Sustainability of public health programming necessitates the capacity to maintain innovations in policy and practice that encourage healthful behaviors. Sustainability is a critical but often unattainable goal of program planning and implementation [1–4]. Research suggests that childhood obesity can be stemmed and even reversed [5–7]. However, the sustained success of these promising interventions has been difficult to maintain in a public health context. Most effective intervention strategies are tested under tightly-controlled conditions that are not easily maintained or scalable after study conclusion [8–11]. In answer to this conundrum, the field of implementation science has begun to investigate a broad range of factors that might influence the potential for sustainability. Guidelines have been developed for evaluating the process of interventions and their implementation [12]. Yet, few studies have developed a priori frameworks with objectively operationalized constructs for sustainability beyond the intervention [13, 14].

Determining the potential for sustainability of new interventions requires first identifying clear advantages (efficacy and efficiency) over existing strategies for organizations and individuals [1, 3, 15]. These innovations must be integrated within organizational structures without unnecessary complexity or burden. Political, organizational and social institutions must support and enhance innovations, and innovations must “fit” culturally, technologically, and temporally. Researchers have begun to develop frameworks [16] and develop recommendations [17] to guide this arena of implementation science, but few constructs have been operationalized for clear measurement [18–21]. Moreover, studies that have begun to operationalize constructs are typically not guided by theoretical frameworks [17, 19, 21]. Most studies do not use the same constructs to measure sustainability across multidimensional projects and health innovations (i.e., multiple sites, sectors or interventions) [17, 20, 22–25].

The Childhood Obesity Research Demonstration (CORD) project provided an opportunity to test a comprehensive, a priori approach for developing a strategy to investigate the potential for sustainability. The CORD intervention framework was derived from the Obesity Chronic Care Model [26–33], including multiple sites, sectors and approaches in the study of childhood obesity prevention and management. The Obesity Chronic Care Model [28] employed an integrated framework focusing on simultaneously changing policies, systems, and environments in healthcare, public schools, early care and education centers, and other community settings [15, 33, 34]. Because of the inherent complexity, we relied on the Ecologic Model of Obesity [35–38] to guide the framework development. The Ecologic Model of Obesity [36] is a dynamic systems framework. We operationalized the constructs of the potential for sustainability across ecologic levels, from macro-level policies to micro-level settings and individual behaviors [35]. Development of this theoretically guided framework helped researchers to make consistent conclusions and recommendations, drawing on mixed methodological approaches.

This manuscript describes the rationale, process, model and resulting methodology from developing the framework and evaluation plan for the cross-site sustainability of CORD. CORD investigated primary and secondary prevention interventions integrating primary care and public health strategies in eight communities in California, Massachusetts, and Texas. CORD aimed to support individual- and family-level behavioral change to prevent and reverse obesity among low-income and racial/ethnic minority children 2–12 years of age [26, 27, 29–32]. For the sustainability evaluation, we constructed a practical framework to describe four dimensions: replicability, continuation of benefits, institutionalization, and community capacity. This manuscript presents the process of developing the framework and a set of lessons learned that may guide future intervention planning and programming to enhance sustainability of these efforts.

## Method

### The CORD project

CORD included three demonstration projects and an evaluation center [26, 27, 29–33]. Each CORD demonstration project team individually developed its own multi-part interventions aimed at simultaneously changing policies, systems, and environments in primary care clinics, public schools, early care and education centers, and community institutions. Each project was guided by the Obesity Chronic Care Model [28] and focused on four health behaviors associated with obesity prevention and control: physical activity, dietary habits, screen time, and sleep. Intervention activities were concurrent across sectors using community health workers to enhance robustness of effects.

The evaluation center comprised a team of investigators charged with collecting and analyzing data across all sites to produce a comprehensive implementation, process and sustainability evaluation [31, 39]. The evaluation center worked collaboratively with the academic and community partners from the three sites together with the funding agency personnel throughout the project via semi-monthly phone calls. The evaluation center was arranged into several workgroups, including implementation, process and sustainability. The sustainability workgroup was comprised of four PhD level investigators and also included four participating trainees. Trainees were PhD and masters students under the mentorship of the investigators. The team had broad training and expertise including behavioral science, civil engineering, geographic information systems, kinesiology, nutrition, obesity research, public policy, research methodology, and social work. Two investigators had advanced training and expertise in measurement development. Two investigators had advanced training and expertise in qualitative research methodology. All four investigators had advanced training in quantitative research methodologies, including one with advanced spatial analysis techniques. The workgroup met weekly to develop and implement the sustainability evaluation plan using data from the three demonstration projects. The entire cross-site evaluation plan was approved by the University of Houston Committee for the Protection of Human Subjects.

### Development of sustainability evaluation plan

The principal challenges in designing the CORD cross-site Sustainability evaluation plan were twofold. First, the inherent complexity of determining sustainability of a multisite project with multiple sectors and protocols required innovative thinking to develop and operationalize a guiding framework. Second, examining the potential for sustainability required a certain amount of forecasting, since sustainability represents a future development. To develop the sustainability evaluation plan, in light of these challenges, investigators followed a systematic, 3-stage approach:

### STAGE 1: identify the goals of the sustainability evaluation workgroup

The workgroup met weekly to define its goals based on the initial CORD proposal. The workgroup needed to identify a set of objective measures that could be consistently collected across all projects. The workgroup sought to find rigorous measures while limiting additional resources spent on collecting sustainability data, as these were not necessarily budgeted into the original scope of work for each site. A second goal of the workgroup was to identify the goals of the CORD Sustainability evaluation plan itself. The last goal was to operationalize the evaluation plan with constructs that could be measured. As time went on, the workgroup developed a fourth goal of collecting some measures that could not be gleaned from the sites themselves.

### STAGE 2: identify the goals of CORD sustainability evaluation

In order to identify the goals of the Sustainability evaluation, the workgroup reviewed the existing literature. The workgroup identified a guiding framework, the Ecologic Model of Obesity [36]. The Ecologic Model of Obesity [36] was developed from the Ecologic Model of Physical Activity (EMPA) [35, 38]. Second, the workgroup linked constructs of sustainability to levels within the framework.

### STAGE 3: operationalize the sustainability evaluation plan

The last stage of the plan was to operationalize the constructs into a standardized set of objective measures at each level of the Ecologic Model of Obesity [36] that might be available consistently across all projects. After the sustainability workgroup had identified the Ecologic Model of Obesity [36] as a guiding framework and the sustainability constructs, discussion and brainstorming were used to identify possible measurement strategies. As described in the Results section, the workgroup identified or developed measures using checklists, existing datasets, or qualitative interviews in order to further measure operationalized constructs. The workgroup also relied on measures that had already been collected by each of the CORD sites whenever possible.

### Measures

*Checklists* were created to count and code various indicators of sustainability operationalized constructs. First, *Program Components Checklists* were created for each site, organized by sector (clinic, school, early care and education, and community) and broken into categories of policies, systems, environments and practices. These categories were further divided into intervention- or measurement-related items for a total of approximately 120 items that could be checked off per site. The workgroup reviewed documentation from each site to create an individual Program Components Checklist for each site. Documentation included the original proposals, meeting minutes, and revised workplans. Examples of items that were included in the Program Components Checklists included training sessions, advocacy efforts, intervention materials (e.g., handouts, manuals, signage), branded promotional materials (e.g., water bottles, t-shirts), counseling sessions, meetings, surveys, and anthropometric measures. Principal investigators for each site were asked to complete one Program Components Checklist to verify that all items that had been completed at each site.

Two *Policy Indicator Checklists* that have been previously published and described in detail were developed for use in this study [40, 41]. Both measured the policies about consumption of high fat foods and sugar sweetened beverages or physical activity in the CORD sectors of clinic, school, early care and education and community settings. The Policy Indicator Checklists were designed to be completed by the sustainability workgroup members by scanning websites concerning policy across the sectors in the CORD project.

*Existing datasets* were identified by the workgroup following the suggestion of our team experts or via internet searches. During workgroup meetings, well established datasets that were already geocoded and publicly available (e.g., US Census) were discussed as possible sources of sustainability data. Workgroup members conducted searches using key words based on the sustainability constructs and their possible indicators such as health system utilization, obesity-related behavior, and health outcomes. Snowball searches were also used after possible candidate datasets had been identified during meeting discussion or from earlier searches. The workgroup discussed the benefits and limitations of all candidate datasets before selecting options for data extraction. Datasets that were selected had to be free for use, publicly accessible, geocoded to the finest resolution possible (e.g., zip code, census tract), and provide information about communities that would be helpful in forecasting the potential for sustainability of interventions.

*In depth interviews* were conducted with principal investigators and key informants at each of the three sites to understand community- and collaboration-level factors that might contribute to sustainability. Principal investigators identified key informants across the CORD sectors. An interview guide was developed along with a carefully constructed telephone interview protocol designed to be conducted by a third party researcher experienced in qualitative interview administration. One investigator experienced in qualitative research methodology constructed an investigator interview guide that included twelve open-ended questions related to constructs of replicability, continuation of benefits, institutionalization, and community capacity, following the levels of the Ecologic Model of Obesity [36] and sustainability constructs. A second investigator experienced in qualitative research reviewed the initial guide, and feedback was incorporated. The penultimate interview guide was reviewed by the entire workgroup, and minor changes incorporated. Examples of questions included “Tell me about the most important existing resources that your site used to integrate CORD program components and delivery?” and “What are your site organizations’ future plans for continuing activities similar to those in CORD?”

## Results

### The guiding framework and constructs of sustainability

**Fig. 1:**
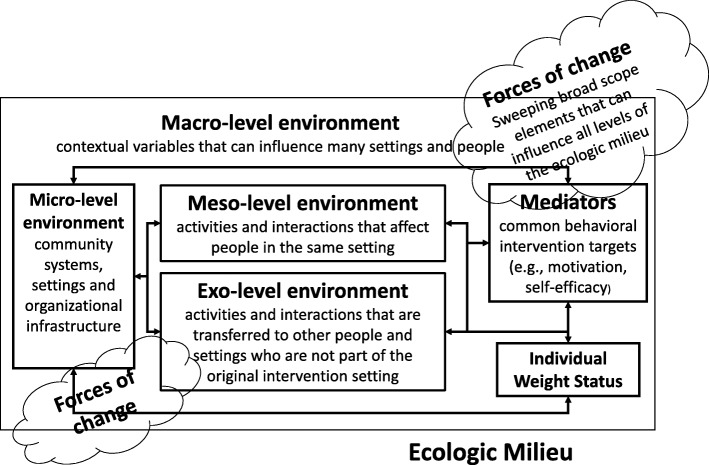
Adapted Ecologic Model of Obesity

The multidimensional, ecologic nature of sustainability necessitated a dynamic systems framework. We examined dimensions influencing the sustainability of childhood obesity interventions within all levels of the Ecologic Model of Obesity [36]. The Ecologic Model of Obesity [36] has demonstrated utility and innovation as a systems framework by describing ecological levels, as well as, dynamic intervention linkages and pathways. These linkages and pathways describe how intervention strategies may reach settings and individuals beyond those for which they were originally intended [35, 37, 38]. Figure 1 illustrates how the levels of the Ecologic Model of Obesity [36] are hypothesized to operate.

We developed sustainability constructs and their measurement by Ecologic Model of Obesity [36] levels—micro-, meso-, exo- and macro-level environments, which interact and influence individual behavior choices. Micro-level environments refer to community systems, settings and organizational infrastructure. Meso-level environments are characterized by direct social and physical dynamic linkages and processes. Meso-level environments are the intervention activities and interactions that are affecting people in the same setting, such as a clinic or school. In contrast, exo-level environments are characterized by *indirect* social and physical dynamic linkages and processes. These are the intervention activities and interactions that are transferred to other people and settings who are not part of the original intervention setting. For example, activities happening at school with a child may indirectly influence choices made by a parent at work—a setting that does not involve the original intervention setting or person (in this case, the school and the child). Macro-level environments define contextual variables that can influence many settings and people, like policies and cultural factors. Forces of change, such as changes in population composition, innovations in social networking and mobile health technology, and globalization, exert pressure on all levels of the model.

To further advance the application of the Ecologic Model of Obesity [36], we integrated sustainability constructs along four dimensions: *replicability*, *continuation of benefits*, *institutionalization* and *community capacity* [3, 31]. Constructs were operationalized by the levels of the Ecologic Model of Obesity [36]. Examples of construct operationalization and measurement are described below and summarized in Table 1.

**Table 1:** Operationalized Sustainability Construct Measurement Sources in the Childhood Obesity Research Demonstration by Ecologic Model of Obesity Levels

Ecologic Model of Obesity Levels	Sustainability Constructs			
	Replicability (intervention complexity, use of existing resources and systems, and adaptability of program activities)	Continuation of Benefits (linkages and communication within organizations, continued infrastructure and resources)	Institutionalization (ongoing integrated support for projects and diffusion of program activities among organizations, policies and practices)	Community Capacity (sense of community, community commitment, problem-solving mechanisms, and resource access)
Micro-level	• Program Components Checklist	N/A	• Program Components Checklist • Health Care Clinic Scan • School and Early Care and Education Center Indices • In-depth Interviews	• Wilder Collaboration Factors Inventory • Geographically Linked, Publicly-available Databases
Meso-level	• Program Components Checklist • Electronic Health Record	N/A	• Wilder Collaboration Factors Inventory • In-depth Interviews • Health Care Clinic Scan • School and Early Care and Education Center Indices	• Wilder Collaboration Factors Inventory • In-depth Interviews
Exo-level	• Program Components Checklist • Electronic Health Record	• Community Health Worker Mobile Device Data	• In-depth Interviews • Health Care Clinic Scan • School and Early Care and Education Center Indices	• Wilder Collaboration Factors Inventory • In-depth Interviews
Macro-level	• In-depth Interviews	• Geographically Linked, Publicly-available Databases	• Policy Indicator Checklists • In-depth Interviews • School and Early Care and Education Center Indices	• Policy Indicator Checklists • Geographically Linked, Publicly-available Databases

### Operationalizing the potential for sustainability constructs

*Replicability* was operationalized as the potential for programs or interventions to be repeated or reproduced partially or fully. Operational indicators of replicability include intervention complexity, use of existing resources and systems, and adaptability of program activities of the interventions used at the three sites of CORD. These are measured within micro-level settings, or as meso- or exo-level connections between people and places [31, 36, 38]. Less complex programs rely on existing resources and systems and require minimal or low cost adaptation. Less complex programs are generally easier to replicate than those that are more complex or rely on new resources and more funding.

To measure *replicability*, we first developed the *Program Components Checklist* (described in Method) [31]. The Program Components Checklist enumerated each site’s intervention activities through reviews of project proposals, project meeting minutes, and other documents. Sites’ principal investigators and project directors verified completed Program Components Checklists to confirm accuracy and identify missing activities. Activities included micro-level strategies targeting specific settings and others providing direct (meso-level) or indirect (exo-level) linkages to shape providers’ and participants’ behaviors. In general, more activities signify increasing complexity, because the effort and resources needed to replicate multicomponent interventions would be expected to increase.

Another measure of replicability is the consistency of *electronic health record* used within clinics, which connects experiences among different clinic visits. Electronic health record data were tabulated to determine consistency of referral as meso-level and exo-level replicability measures. Consistent electronic health record use indicates what happens in subsequent visits to the same clinic (meso-level). It also measures indirect linkages with other settings or providers (exo-level), such as community health workers. Identifying and measuring these direct and indirect pathways is fundamental to the Obesity Chronic Care Model [28].

We used *in-depth interviews* with key informants in clinical, early care and education center, school and community settings to measure macro-level factors. Interviews ask open ended questions about alignment among the agendas of providers, organizations, and government agencies, as this may indicate the existence of important supports such as political will and health-related social norms, macro-level factors.

*Continuation of benefits* is a construct derived from the health insurance industry, where it refers to coverage aspects that remain after lapses in work from disability, illness or retirement [42]. In the broader context of sustainability of public health programming, continuation of benefits refers to the maintenance of program effectiveness [31]. Continuation of benefits framed in the Ecologic Model of Obesity [36] include improved linkages and communication within organizations (meso-level). Also, benefits include infrastructure (micro- and macro-level), and resources (greater funding, more staffing, safer and more functional buildings—micro-level) to complete mission-derived programming. Continuation of benefits could be compromised by loss of providers or organizations. In contrast, greater adherence to program activities by providers (meso-level improvements) and expanding reach beyond initial catchment areas or target population (micro- or macro-level improvements) increases the continuation of benefits [35, 36, 38].

Our time frame to measure continuation of benefits was limited to the period of implementation. We were unable to document continued longer-term benefits to end users, the participating children and families, within the project period. Thus, we focused on documenting external, exo- and macro-level factors that would be expected to contribute to longer-term benefits. For example, exo-level linkages included community health workers’ ability to link child and family information between providers. We proposed to count and code access to and use of secure mobile devices and apps by using site collected *community health worker mobile device data*.

To measure macro-level continuation of benefits, we linked project communities to data from *geographically linked, publicly-available databases* on health system utilization, obesity-related behavior and health outcomes (e.g., American Community Survey, Environmental Systems Research Institute Business Analyst, Behavioral Risk Factor Surveillance System, WalkScore database) [43–46].

*Institutionalization* is the result of the full integration of a successful program, intervention, or innovation into an existing system [3, 47]. Institutionalization means introducing and implementing an activity, custom, practice or rule and the supports necessary to maintain it in a place, system or organization. Institutionalization is the result of the adoption and incorporation of specific organizational structures and formal systems to maintain consistent practices or patterns of desirable activities, behaviors, or processes. Measuring institutionalization involves examining organizations (micro-level settings), practices (meso- and exo-level linkages) and policies (macro-level influences) that support or enforce program activities or systems [35, 36, 38].

Measuring *institutionalization* included indicators of ongoing, integrated support for projects or oversight and diffusion of program activities among organizations, policies and practices across the levels of the Ecologic Model of Obesity [36]. We measured environments and practices from participating clinics, early care and education centers, and schools prior to implementation and again after 24 months of implementation. Improvements in these measures define improvements in institutionalization.

For clinics, we used data from a multi-part *Health Care Clinic Scan* to assess systems and environmental changes associated with health behavior improvements. Clinic administrators from CORD sites completed the survey portion of the Scans about patient volume, staffing, and standard operating procedures believed to influence institutionalization of effective strategies to promote healthy eating and physical activity. CORD site teams audited clinics for resources supporting healthy food options (e.g., vending machines, messaging, publicly available handouts) and active transportation options (e.g., biking, walking, public transportation) for families and staff. Last, physician champions completed surveys about clinical information systems, changes to the clinic delivery system, and decision supports in places to aid providers in assessing and managing children’s weight status.

We used a composite *School and Early Care and Education Center Index* for early care and education centers and schools about health policies and practices. Composite indices were administered by CORD site teams and comprised of items from validated instruments such as the Nutrition and Physical Activity Self-Assessment for Child Care [48], Alliance for a Healthier Generation Healthy Schools Inventory Worksheet [49], School Physical Activity and Nutrition survey [50], and site-developed school leader surveys. These included items about nutrition education, physical activity, water and calorically sweetened beverage consumption, and general child wellness. We matched and standardized common measures across projects to produce a single score summarizing the aggregate impact of programs previously shown to be effective in influencing key behaviors.

The sustainability team developed the *Policy Indicator Checklists* to document the macro-level policy environment governing community settings [40, 41]. The first identifies and measures policies for calorie-dense foods and sugar-sweetened beverages, and the second for physical activity. The checklists define and document policies across early care and education center, school, and community settings for an entire community. We used these checklists to measure institutionalization within micro-level healthcare, school or community settings [31]. Micro-level policies as measured by these checklists may lead to consistent training protocols and continuing education. Training resources can foster meso-level communication between clinicians and consumers and exo-level communication between consumers and their friends and family, which enhance institutionalization.

In order to measure longer-term indicators of institutionalization and provide deeper understanding we relied on responses to *in-depth interviews* (as initially described above). These interviews included open-ended questions to document and understand institutionalization across ecologic levels that may not have been captured by other proposed measures.

The concept of *community capacity* emerged in the 1990s via economic development and has been applied to numerous public health challenges. Community capacity involves engaging resources to complete collective goals [51]. Community capacity comprises formal and informal organizations and alliances that can influence sustainability via social processes (meso- and exo-level linkages like coalition building and social networking) and physical structures (micro-level elements like parks or schools) [35, 36, 38, 52]. Community capacity is operationalized as sense of community, community commitment, problem-solving mechanisms, and resource access. Community capacity measures the collective human, social and physical assets of communities that are available to sustain and expand programming and services [52].

*Community capacity* appears—by virtue of the word, “community”—to be a macro-level construct, but components of it can be measured at all levels of the Ecologic Model of Obesity [36]. For example, the presence of public spaces like schoolyards, parks and other shared resources provide micro-level places for people to engage in physical activity and interact socially, which increases community capacity. Transportation networks can provide exo- and meso-level linkages between micro-level settings and meso-level communication via visual marketing materials and auditory behavioral prompts.

We linked data from *publicly-available databases* (e.g., community websites for parks and recreation and geographic information systems departments and WalkScore) to measure the existence and stability of community goods, services, and resources. For example, data from the WalkScore database were used to assess the availability of physical activity and food resources in neighborhoods surrounding schools and early care and education centers in project communities. These databases provided both micro-level and macro-level environment information about community capacity.

Exo-, meso-, and micro-level community supports were measured via the *Wilder Collaboration Factors Inventory*. The Wilder measures the level of collaboration along six dimensions: collaboration purpose, member characteristics, communication, process/structure, environment and resources [53]. The Wilder was administered to representatives on advisory boards that planned and informed CORD interventions from various settings and institutions. These included schools and school districts, early care and education centers, clinics and healthcare systems, and health and public safety departments. An example of meso-level community support is the extent to which coalition members adopt and work toward shared goals and vision. A micro-level example is the degree of group cohesion that helps make innovations sustainable.

We relied on the *Policy Indicator Checklists* to measure macro-level state and local policies supporting healthful behaviors as indicators of both community capacity [40, 41]. Macro-level policies may help enforce programming and individual behavior. In-depth interviews rounded out the information collected to operationalize community capacity.

## Discussion

Implementation of the theoretically-guided, and distinctly operationalized measures from our evaluation of the potential for sustainability of CORD projects varied. We succeeded in some cases but were unable to overcome some barriers in this large and complex multisite project, learning the following key lessons summarized in Table 2.

**Table 2:** Challenges, lessons learned and possible solutions in the development of the CORD Sustainability Evaluation

*Challenge*	*Lesson*	*Solution*
1. Planning vs. Implementation	Investigators must rely on observations of implemented activities, rather than merely considering planned activities.	Use easy-to-complete checklists to verify planned activities and programming.
2. Competing Priorities and Expectations	Complex projects should have collaboratively designed common measures of sustainability constructs prior to implementation	Funding announcements, agencies and collaborative teams should specify a priori sustainability measures that must be completed as part of the protocol.
3. Flexibility in Measures	Constructs should be clearly defined so that they may be measured using multiple strategies (e.g., direct observation, qualitative approaches, review of public datasets)	Have strong and clearly defined set of theoretically constructs that can be measured in multiple ways.
4. Site and Community Communication	Strong and established communication channels linking investigators and community representatives can facilitate evaluation of the community context influencing projects	Implement communication strategies that involve investigators, implementers and data collectors to build relationships that enhance data collection.
5. Publicly Available Data Sets	Triangulate among public data sources to enhance data consistency and scientific rigor.	Verify and identify redundant public data sets prior to beginning the study to improve validity of public data.
6. Labor Intensive Evaluation Processes	Sustainability variables should be planned and adequately budgeted prior to the start of implementation and programming.	Include the execution of the sustainability plan into initially budgeted activities as specified by funding announcements or agencies.

### Lesson 1. Planning vs. implementation

It became evident early that proposed plans were not necessarily accurate descriptions of what ultimately unfolded. Demonstration project sites had ambitious and multi-level intervention plans, as required by the funding opportunity announcement, and provisions in implementation plans accommodated setting-specific needs. In some cases implementation strategies differed from what was planned due to unanticipated barriers (e.g., leadership and policy changes). Some implementation strategies were modified substantially to accommodate resource or time limitations (e.g., available training time for teachers in school-based interventions). The Program Components Checklist was necessary to obtain accurate measures comparing plans with implementation. In order to accurately track planning and implementation of various aspects of large-scale interventions for assessing complexity and replicability, site-specific checklists should be created and verified during the early stages of intervention development. And, these checklists may need to evolve as implementation plans become finalized. This process should involve close collaboration between researchers from the evaluation or coordinating center and researchers from intervention sites with knowledge of all project aspects.

### Lesson 2. Competing priorities and expectations

Scientists are smart, strong willed, courageous, and highly value autonomy and control over their work. Expecting four teams of scientists to adapt data collection efforts to match a common set of measures, potentially requiring substantial revisions to original plans and altering agreements with community partners, was challenging for everyone involved. More clearly defined rationale, consensus priorities, and explicitly stated expectations from the beginning of the project might have eased data collection regarding sustainability constructs. Sites had to emphasize project start up and implementation, with sustainability evaluation as a secondary priority. A project that is not implemented cannot be sustained. Childhood obesity interventions that are intended to be real-world in implementation, like the CORD projects, do not occur under tightly controlled experimental conditions. Competing priorities, funding limits and pressures, and time constraints made it difficult to coordinate a large set of common measures. Future, multi-site, collaborative projects should identify and agree upon cross-site sustainability constructs and measures from the beginning to obtain robust and informative data.

### Lesson 3. Flexibility in measures

As the sustainability evaluation plan emerged, it became evident that operationalizing constructs would require flexibility, particularly since most data collection was to be conducted by the site investigators rather than the evaluation center itself. For example, a quantitative questionnaire was developed to obtain information from key informants and providers at each project, but this was ultimately unfeasible for individual sites because of staff time constraints and impeding priorities. Thus, we used different approaches to obtain data: qualitative interviews and publicly available datasets. It was important to have strong and clearly defined constructs that could be measured in multiple ways and then triangulated to obtain data on all constructs and maintain scientific rigor.

### Lesson 4. Site and community communication

One unexpected challenge was coordinating communication across communities, likely due to the complexity and scope of projects and evaluation plans. Facilitated by twice monthly conference calls and annual, face-to-face meetings and site visits, communication among projects’ principal investigators developed strongly. However, communication of messages from the evaluation center across the participating communities varied. For example, the evaluation center planned a survey for administration to city government personnel to answer questions about community planning efforts and policies supporting physical activity and healthy eating for children. The site institutions and investigators had ongoing relationships with those personnel, and site investigators were asked to coordinate communication between city personnel and the evaluation center to complete the survey. Although some city personnel completed sections, obtaining complete responses was limited by the indirect communication route from the evaluation center, through the sites, to the respective city personnel. Stronger linkages between investigators and community representatives might have enhanced sustainability data collection and facilitate evaluation of the community context influencing projects.

### Lesson 5. Publicly available data sets

Another challenge faced in the operationalization of constructs was reliance on geographically-linked publicly available data sets. These data sets were necessary in some cases, since in-person, full audits by the evaluation center staff of each community’s environment and resources were unfeasible. The availability of updated data often differed between municipalities and between city, county, and state levels. The precision and accuracy of the data were sometimes unclear. This challenge may have resulted from different priorities or available funding in each community to collect, maintain, and update geographic information systems data, as previously reported [54]. For example, some communities had extensive, geocoded data sets showing locations and sizes of parks or lengths of walking and bicycling trails, whereas other communities had less detailed information available. Further, the accuracy of these sources was frequently unclear, and the years in which sources were updated were inconsistent. It became clear that collecting and analyzing data about community context necessitated triangulation among sources such as community websites, geographic information systems datasets, and Google Maps Street View.

### Lesson 6. Labor intensive evaluation processes

As a result of obstacles discussed previously, some of the measures (e.g., Program Components Checklists, Policy Indicator Checklists) that were used to collect data required de novo development with supplementation from other data. This development required significant labor and time. This effort was unanticipated and outside of the primary evaluation design and data collection plan for each site. With stronger initial planning for sustainability evaluation, we may have been able to integrate our questionnaires into the primary evaluation and data collection plans for each site. Future studies should begin with a well specified sustainability evaluation plan from the start and should budget accordingly for staffing to collect data as needed.

Although previous efforts to enhance sustainability have been developed for practitioners (e.g., Sustainability Planning Guide) [55] there has been relatively little theoretically integrated work defining and operationalizing constructs a priori to enhance funded research projects [13, 14]. The present work yielded important lessons and required innovation, creativity, and flexibility to obtain data fitting our operationalized constructs. The Ecologic Model of Obesity [36] was applied to guide operationalization of sustainability constructs to develop a viable mixed-method process for evaluating sustainability. Our team developed measures and processes to apply valid and reliable, existing and newly collected, quantitative and qualitative methods for the project to account for discrepancies among data sources. Future research should continue to test these measures and translate their findings to inform policy and practice and guide practical implementation and sustainability efforts. The needs for intensive, advanced planning; high levels of communication among researchers and providers; and inclusion of reliable and valid measures; before program implementation were important lessons learned from creating the sustainability evaluation in this project. The importance of aligning priorities and expectations for sustainability evaluation also became clear, particularly for multisite projects.

## Conclusion

This manuscript describes the rationale, process, model and resulting methodology involved in operationalizing a practical framework to describe the four dimensions of replicability, continuation of benefits, institutionalization, and community capacity of the sustainability evaluation plan of the CORD project. Measuring sustainability of multilevel, multisite interventions remains complex, although the work described herein provides a bridge to begin to understand how sustainability constructs can be applied practically. These findings may inform future research and practice using sound evidence that is consistently defined, collected, and reported.

## Data Availability

Not applicable.

## References

[1] Huang TTK, Grimm B, Hammond RA (2011). A systems-based typological framework for understanding the sustainability, scalability, and reach of childhood obesity interventions. Child Health Care.

[2] Pluye P, Potvin L, Denis JL (2004). Making public health programs last: conceptualizing sustainability. Eval Program Plann..

[3] Shediac-Rizkallah MC, Bone LR (1998). Planning for the sustainability of community-based health programs: conceptual frameworks and future directions for research, practice and policy. Health Educ Res.

[4] Simmons A, Borys JM, Swinburn B, Waters E, Swinburn BA, Seidell JC, Uauy R (2010). Community interventions—planning for sustainability. Preventing childhood obesity: evidence policy and practice.

[5] Economos CD, Curtatone JA (2010). Shaping up Somerville: a community initiative in Massachusetts. Prev Med.

[6] Epstein LH, Valoski A, Wing RR, McCurley J (1994). Ten-year outcomes of behavioral family-based treatment for childhood obesity. Health Psychol.

[7] Quattrin T, Roemmich JN, Paluch R, Yu J, Epstein LH, Ecker MA (2012). Efficacy of family-based weight control program for preschool children in primary care. Pediatrics..

[8] Huang TT, Drewnosksi A, Kumanyika S, Glass TA (2009). A systems-oriented multilevel framework for addressing obesity in the 21st century. Prev Chronic Dis.

[9] Summerbell CD, Waters E, Edmunds LD, Kelly S, Brown T, Campbell KJ. Interventions for preventing obesity in children. Cochrane Database Syst Rev. 2005;(3):Cd001871. 10.1002/14651858.CD001871.pub2 16034868

[10] Wang Y, Beydoun MA (2007). The obesity epidemic in the United States--gender, age, socioeconomic, racial/ethnic, and geographic characteristics: a systematic review and meta-regression analysis. Epidemiol Rev.

[11] Wang Y, Cai L, Wu Y, Wilson RF, Weston C, Fawole O (2015). What childhood obesity prevention programmes work? A systematic review and meta-analysis. Obes Rev.

[12] Moore GF, Audrey S, Barker M, Bond L, Bonell C, Hardeman W (2015). Process evaluation of complex interventions: Medical Research Council guidance. BMJ..

[13] Hailemariam M, Bustos T, Montgomery B, Barajas R, Evans LB, Drahota A (2019). Evidence-based intervention sustainability strategies: a systematic review. Implement Sci.

[14] Wiltsey Stirman S, Kimberly J, Cook N, Calloway A, Castro F, Charns M (2012). The sustainability of new programs and innovations: a review of the empirical literature and recommendations for future research. Implement Sci.

[15] Johnson K, Hays C, Center H, Daley C (2004). Building capacity and sustainable prevention innovations: a sustainability planning model. Eval Program Plann.

[16] Schell SF, Luke DA, Schooley MW, Elliott MB, Herbers SH, Mueller NB (2013). Public health program capacity for sustainability: a new framework. Implement Sci.

[17] Grol RP, Bosch MC, Hulscher ME, Eccles MP, Wensing M (2007). Planning and studying improvement in patient care: the use of theoretical perspectives. Milbank Q.

[18] Damschroder LJ, Aron DC, Keith RE, Kirsh SR, Alexander JA, Lowery JC (2009). Fostering implementation of health services research findings into practice: a consolidated framework for advancing implementation science. Implement Sci.

[19] Eccles MP, Armstrong D, Baker R, Cleary K, Davies H, Davies S (2009). An implementation research agenda. Implement Sci.

[20] Scheirer MA (2005). Is sustainability possible? A review and commentary on empirical studies of program sustainability. Ame J Eval.

[21] Squires JE, Estabrooks CA, O'Rourke HM, Gustavsson P, Newburn-Cook CV, Wallin L (2011). A systematic review of the psychometric properties of self-report research utilization measures used in healthcare. Implement Sci.

[22] Chaudoir SR, Dugan AG, Barr CH (2013). Measuring factors affecting implementation of health innovations: a systematic review of structural, organizational, provider, patient, and innovation level measures. Implement Sci.

[23] Gruen RL, Elliott JH, Nolan ML, Lawton PD, Parkhill A, McLaren CJ (2008). Sustainability science: an integrated approach for health-programme planning. Lancet..

[24] Mayer AL (2008). Strengths and weaknesses of common sustainability indices for multidimensional systems. Environ Int.

[25] Rychetnik L, Frommer M, Hawe P, Shiell A (2002). Criteria for evaluating evidence on public health interventions. J Epidemiol Community Health.

[26] Ayala GX, Ibarra L, Binggeli-Vallarta A, Moody J, McKenzie TL, Angulo J (2015). Our choice/Nuestra Opcion: the Imperial County, California, childhood obesity research demonstration study (CA-CORD). Child Obes.

[27] Davison KK, Falbe J, Taveras EM, Gortmaker S, Kulldorff M, Perkins M (2015). Evaluation overview for the Massachusetts childhood obesity research demonstration (MA-CORD) project. Child Obes.

[28] Dietz WH, Solomon LS, Pronk N, Ziegenhorn SK, Standish M, Longjohn MM (2015). An integrated framework for the prevention and treatment of obesity and its related chronic diseases. Health Aff (Millwood).

[29] Foltz JL, Belay B, Dooyema CA, Williams N, Blanck HM (2015). Childhood obesity research demonstration (CORD): the cross-site overview and opportunities for interventions addressing obesity community-wide. Child Obes.

[30] Hoelscher DM, Butte NF, Barlow S, Vandewater EA, Sharma SV, Huang T (2015). Incorporating primary and secondary prevention approaches to address childhood obesity prevention and treatment in a low-income, ethnically diverse population: study design and demographic data from the Texas childhood obesity research demonstration (TX CORD) study. Child Obes.

[31] O'Connor DP, Lee RE, Mehta P, Thompson D, Bhargava A, Carlson C (2015). Childhood obesity research demonstration project: cross-site evaluation methods. Child Obes.

[32] Taveras EM, Blaine RE, Davison KK, Gortmaker S, Anand S, Falbe J (2015). Design of the Massachusetts Childhood Obesity Research Demonstration (MA-CORD) study. Child Obes.

[33] Williams N, Dooyema CA, Foltz JL, Belay B, Blanck HM (2015). The childhood obesity research demonstration project: a team approach for supporting a multisite, multisector intervention. Child Obes.

[34] Dietz W, Lee J, Wechsler H, Malepati S, Sherry B (2007). Health plans' role in preventing overweight in children and adolescents. Health Aff (Millwood)..

[35] Lee RE, Cubbin C (2009). Striding toward social justice: the ecologic milieu of physical activity. Exerc Sport Sci Rev.

[36] Lee RE, Leach HJ, Soltero EG, Vollrath KR, Hallett AM, Parker NH (2015). Obesity: an ecologic perspective on challenges and solutions. Public health nutrition: principles and practice in community and Global Health.

[37] Lee RE, Medina AV, Mama SK, Reese-Smith JY, O'Connor DP, Brosnan M (2011). Health is power: an ecological, theory-based health intervention for women of color. Contemp Clin Trials.

[38] Spence JC, Lee RE (2003). Toward a comprehensive model of physical activity. Psychol Sport Exerc.

[39] Joseph S, Stevens AM, Ledoux T, O'Connor TM, O'Connor DP, Thompson D (2015). Rationale, design, and methods for process evaluation in the childhood obesity research demonstration project. J Nutr Educ Behav.

[40] Lee RE, Hallett AM, Parker N, Kudia O, Kao D, Modelska M (2015). Development of the policy indicator checklist: a tool to identify and measure policies for calorie-dense foods and sugar-sweetened beverages across multiple settings. Am J Public Health.

[41] Lee RE, Hallett AM, Parker NH, Kao D, Modelska MJ, Rifai HS (2015). Physical activity policies in childhood obesity research demonstration (CORD) communities. Health Behav Policy Rev.

[42] Health care continuation coverage--Pension and Welfare Benefits Administration--Department of Labor (1997). Request for information. Fed Regist.

[43] Walk Score. https://www.walkscore.com/. Accessed 10 January 2020.

[44] Centers for Disease Control and Prevention. Behavioral Risk Factor Surveillance System Atlanta, Georgia: U.S. Department of Health and Human Services, Centers for Disease Control and Prevention; http://www.cdc.gov/brfss/. Accessed 10 January 2020.

[45] Environmental Systems Research Institute. ArcGIS Business Analyst. http://www.esri.com/software/businessanalyst. .

[46] United States Census Bureau. American Community Survey (ACS). https://www.census.gov/programs-surveys/acs/. .

[47] Oldenburg BF, Sallis JF, Ffrench ML, Owen N (1999). Health promotion research and the diffusion and institutionalization of interventions. Health Educ Res.

[48] Ammerman AS, Ward DS, Benjamin SE, Ball SC, Sommers JK, Molloy M (2007). An intervention to promote healthy weight: nutrition and physical activity self-assessment for child care (NAP SACC) theory and design. Prev Chronic Dis.

[49] Alliance for Health Generation. Healthy Schools Inventory worksheet. https://schools.healthiergeneration.org/. Accessed 10 January 2020.

[50] Hoelscher DM, Day RS, Kelder SH, Ward JL (2003). Reproducibility and validity of the secondary level school-based nutrition monitoring student questionnaire. J Am Diet Assoc.

[51] Fawcett SB, Paine-Andrews A, Francisco VT, Schultz JA, Richter KP, Lewis RK (1995). Using empowerment theory in collaborative partnerships for community health and development. Am J Community Psychol.

[52] Chaskin RJ (1999). Defining community capacity: a framework and implications from a comprehensive community initiative. Urban Affairs association annual meeting.

[53] Mattessich P, Murray Close M, Monsey B (2001). Wilder collaboration factors inventory.

[54] Hirsch JA, Meyer KA, Peterson M, Rodriguez DA, Song Y, Peng K (2016). Obtaining longitudinal built environment data retrospectively across 25 years in four US cities. Front Public Health.

[55] Batan M, Butterfoss FD, Jaffe A, LaPier T (2012). A sustainability planning guide for healthy communities. Centers for Disease Control and Prevention, National Center for Chronic Disease Prevention and Health Promotion, division of community health, editors.

